# Relationship between maternal obesity and first-trimester TSH in women with negative anti-TPO antibodies

**DOI:** 10.1530/ETJ-23-0213

**Published:** 2024-04-18

**Authors:** Laura Croce, Fausta Beneventi, Federica Ripepi, Irene De Maggio, Alberto Malovini, Camilla Bellingeri, Francesca Coperchini, Marsida Teliti, Mario Rotondi, Arsenio Spinillo, Flavia Magri

**Affiliations:** 1 Department of Internal Medicine and Therapeutics, University of Pavia, Pavia (PV), Italy; 2 Istituti Clinici Scientifici Maugeri IRCCS, Unit of Endocrinology and Metabolism, Laboratory for Endocrine Disruptors, Pavia (PV), Italy; 3 Department of Obstetrics and Gynecology, IRCCS Foundation Policlinico San Matteo, University of Pavia, Pavia (PV), Italy; 4 Istituti Clinici Scientifici Maugeri IRCCS, Laboratory of Informatics and Systems Engineering for Clinical Research, Pavia (PV), Italy

**Keywords:** thyroid, pregnancy, obesity, TSH, hypothyroidism

## Abstract

**Objective:**

Obesity is associated with increased thyroid-stimulating hormone (TSH) in non-pregnant subjects, but this phenomenon has not been fully characterized during pregnancy. Our aim was to evaluate the impact of BMI on first-trimester TSH in a wide cohort of pregnant women with negative anti-thyroperoxidase antibodies (AbTPO) and its implications on uterine artery pulsatility index (UtA-PI), a marker of early placentation.

**Methods:**

The study included 2268 AbTPO-negative pregnant women at their first antenatal visit. Anamnestic data, BMI, TSH, anti-nuclear antibody (ANA) and extractable nuclear antigen (ENA) positivity and mean UtA-PI were collected.

**Results:**

A total of 1693 women had normal weight, 435 were overweight and 140 were obese. Maternal age, ANA/ENA positivity, history of autoimmune diseases and familiar history of thyroid diseases were similar in the three groups. TSH was significantly higher in obese women (1.8 (IQR: 1.4–2.4) mU/L) when compared to normal weight (1.6 (IQR: 1.2–2.2) mU/L) and overweight (median: 1.6 (IQR: 1.2–2.2) mU/L) ones (*P* < 0.001). BMI was significantly related with the risk of having a TSH level ≥4 mU/L at logistic regression, independently from non-thyroid autoimmunity, smoking or familiar predisposition for thyroid diseases (OR: 1.125, 95% CI: 1.080–1.172, *P* < 0.001). A restricted cubic splines regression showed a non-linear relationship between BMI and TSH. Women with a TSH ≥4 mU/L had a higher UtA-PI, independently from BMI.

**Conclusion:**

Overweight/obesity is significantly related with TSH serum levels in AbTPO-negative pregnant women, independently from the other risk factors for hypothyroidism during pregnancy. The increase of TSH levels could be clinically relevant, as suggested by its association with abnormal UtA-PI, a surrogate marker of abnormal placentation.

## Introduction

Subclinical thyroid dysfunction during pregnancy is a frequent condition. The latest American Thyroid Association (ATA) guidelines on thyroid dysfunction during pregnancy revised the TSH serum threshold for the diagnosis of subclinical hypothyroidism (SCH) during pregnancy, raising it up to 4.0 mIU/L, during the first trimester (1). According to this novel TSH threshold, a recent meta-analysis estimated the prevalence of SCH to be around 3% of pregnancies (2). This change in diagnostic criteria is not free of clinical consequences, since it clearly impacted on treatment recommendation by scientific societies. In particular, the latest ATA guidelines suggest different thresholds for initiating levothyroxine (LT4) treatment in pregnant women with or without positive thyroid autoantibodies, while the European Thyroid Association (ETA) guidelines recommend treatment for all pregnant women with SCH (3). The main reason for these disparities derives from lack of clear-cut evidence for the untoward effects of SCH in pregnant women and in their offspring, in particular in women with negative serum anti-thyroid antibodies. While some authors suggest some impact on pregnancy complication and on the neurological development of the offspring in these women (4, 5), other authors did not describe any association (6). In this complicated scenario, identifying any possible cause leading to an increase in serum TSH levels during pregnancy that is not due to thyroid autoimmunity appears worthwhile.

An increased TSH level in the absence of circulating thyroid autoantibodies is a rather frequent finding in the non-pregnant population. Among the possible causes, serum-negative thyroiditis is one of the most prevalent causes, accounting for up to 20% of cases of chronic autoimmune thyroiditis (7), but also laboratory interferences (8, 9) and the presence of TSH genetic variants (10) can explain this phenomenon. Not only moderate–severe iodine deficiency but also iodine excess are linked with higher TSH levels both in non-pregnant subjects (11) and during pregnancy (12). More recently, also a poor maternal iron status was suggested as a cause of higher TSH levels during pregnancy, especially in areas of borderline iodine deficiency (13). Moreover, patients living with morbid obesity frequently experience a raised serum TSH, in the absence of positivity for thyroid autoantibodies and/or abnormal findings at thyroid ultrasound (14). The physiopathology and clinical consequences of this condition have not yet been fully elucidated: some authors suggest that the increase in TSH levels probably does not reflect a hypothyroid state but rather a compensatory ‘hypometabolic’ mechanism due to the high fat mass that quickly recedes after weight loss (14, 15).

Some previous studies on thyroid function during pregnancy observed a positive correlation between TSH levels and BMI during pregnancy (16, 17, 18, 19, 20, 21, 22), while no correlation was observed in other studies (23, 24, 25). Nevertheless, to the best of our knowledge, no specific study assessed the role of BMI in determining TSH levels in pregnant women with negative anti-thyroid antibodies. In a previous study by our group, we observed that pregnant women with negative circulating anti-thyroperoxidase antibodies (AbTPO) and a TSH level ≥4 mU/L had a higher BMI compared with those with a TSH <4 mU/L (5). Moreover, an increase in the occurrence of abnormal uterine artery pulsatility index (UtA-PI) was observed. UtA-PI is a surrogate marker of the quality of the early placentation process. Vasoconstriction of the uterine arteries is a surrogate measure of impaired invasion of spiral arteries by the trophoblast, leading to an impaired fetal growth and higher risk of preeclampsia (26, 27).

The aim of this study was to evaluate in a wide cohort of AbTPO-negative pregnant women the impact of overweight and obesity on serum TSH levels and the possible implications on UtA-PI.

## Subjects and methods

The study included consecutive pregnant women at their first antenatal visit. All women were living in the area surrounding Pavia, Italy, an area characterized by a mild iodine deficiency. Inclusion criteria were: (i) a BMI greater or equal to 18.5 kg/m^2^; (ii) negative test for AbTPO; (iii) availability of serum TSH measurement at the enrollment; (iii) maternal age >18 years; and (iv) singleton pregnancy. Exclusion criteria were: (i) twin pregnancies; (ii) *in vitro* fertilization pregnancy; (iii) known thyroid disorders and/or any medication with an impact on thyroid function; and (iv) positivity for AbTPO. The final study group included 2268 AbTPO negative pregnant women. Given that no trimester-specific range is available for our laboratory, according to their first-trimester serum TSH, pregnant women were stratified according to a TSH value above or below 4 mUI/L, following the recommendations of the latest ATA guidelines (1).

**Figure 1:**
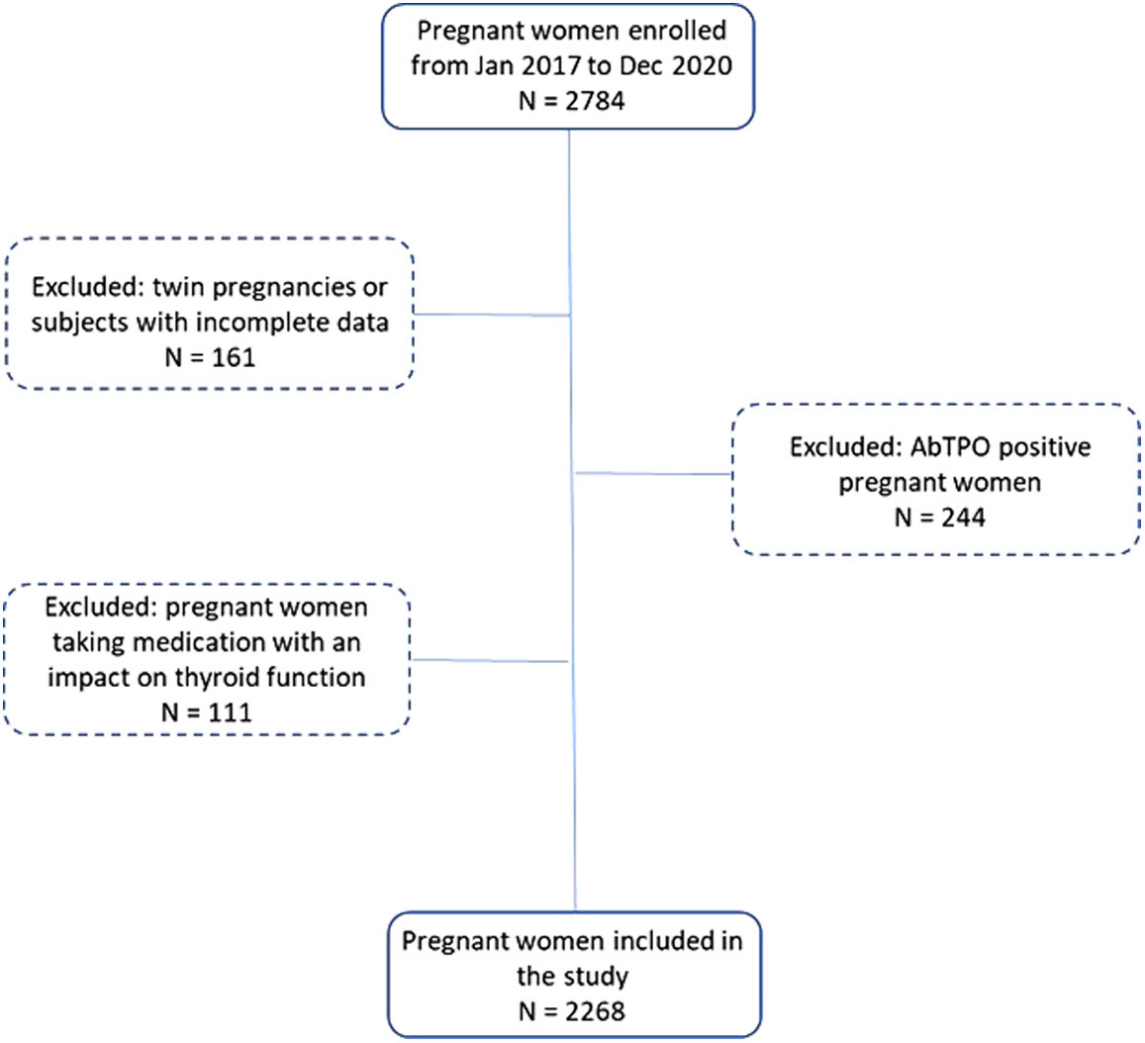
Flow diagram of the study.

The protocol was approved by the Ethics Committee of the Fondazione IRCCS Policlinico San Matteo, Pavia, Italy (901-rcr2017i-23) and performed in accordance with the Helsinki Declaration. All subjects enrolled signed an informed consent concerning the future use of their clinical data for research purposes. Figure 1 shows the flow diagram of the study.

### Data collection

All participants were evaluated during their first antenatal visit (8th–10th gestational week) and completed a medical questionnaire including parity, smoking habit, previous miscarriage, preexisting diagnosis of type 1 diabetes, celiac and rheumatic diseases, and family history for thyroid diseases. Weight and height were assessed and BMI was calculated by dividing the weight by the square of height (kg/m^2^). Maternal BMI 18.5–24.9 identified normal weight women, BMI 25.0–29.9 overweight and BMI >30.0 obese pregnant women.

Blood samples were obtained in fasting condition. The serum concentrations of TSH were measured using a third generation immunoassay Immunolite 2000® Systems Analyzer (Siemens Healthcare) employing monoclonal antibodies. In the absence of a trimester-specific reference range, we chose the reference range for TSH in the non-pregnant adult population, which is 0.4–4.0 mUI/L. The analytic sensitivity of the assay is 0.004 mUI/L. The intra-assay coefficient of variation (CV) ranges from 3.8% to 12.5% and the inter-assay CV from 4.5% to 12.5%. Serum concentrations of antithyroid peroxidase antibody (normal range: ≤40 IU/mL) were measured using Immulite 2000® Anti-TPO Ab, an enzyme-labeled, solid-phase, chemiluminescent sequential immunometric assay (Siemens Healthcare, Erlangen, Germany). The analytic sensitivity of the assay is 5.0 IU/mL. The intra-assay Coefficient of Variation (CV) is 4.9% and the inter-assay CV is 6.3% (28).

In this cohort of pregnant women, ANA and ENA were routinely measured in the context of an ongoing screening for rheumatic diseases in pregnancy (29). ANAs were tested using a standard indirect immunofluorescence technique with a fluorescence microscope (BX 51 Olympus) using a 40× magnification. Sera were first diluted 1:80 in phosphate-buffered saline (PBS) and overlaid onto fixed Hep-2 cell slides (Immuno Concept, Sacramento, CA, USA) at room temperature in a moist chamber for 30 min. Slides were subsequently rinsed and washed in PBS twice for 10 min. As the fluorescent conjugate, a fluorescein-labeled antibody specifically directed toward human immunoglobulin G (IgG) (gchains) (DeltaBiologicals, Pomezia, Italy) was used. All the positive samples (titre > 1:80) were then evaluated at increasing dilutions in PBS (up to 1:640). ENA antibodies were evaluated in triplicate by commercially available ELISA kits (EliA Symphony, Thermo Fisher) according to the manufacture’s recommendations. The following ENA specificities were investigated: Sm, Scl-70, RNP, SSB(La), SSA(Ro) and Jo1 (29, 30).

The mean UtA-PI was measured at first trimester according to standard methods (28, 31). In details, ultrasound studies were carried out by two board-certified operators (F.B. and C.B.). Uterine artery Doppler waveforms were measured after identification of the internal cervical os, with a sampling gate at 2 mm and with an angle of insolation <30°. PI were measured after obtaining three similar consecutive waveforms. PI of uterine arteries were considered abnormal when values were higher than the 95th percentile of reference curves (32).

### Statistical analysis

Statistical analysis was performed using the SPSS software (SPSS, Inc., Evanston, IL) except when otherwise specified. Continuous variables are expressed as median (25th–75th percentiles). Qualitative data were expressed as frequencies. Comparison between groups was performed using the *χ* ^2^-test with Fisher’s correction for frequencies and the Mann–Whitney (in case of two group comparisons) or Kruskal–Wallis (for more than two group comparisons) tests for continuous variables. The strength of the correlation between continuous variables was quantified in terms of Spearman correlation coefficient. A multivariable logistic regression model was designed including a TSH ≥4 mU/L as a dependent variable and BMI (kg/m^2^), non-thyroid autoimmunity (i.e. personal history of a non-thyroidal autoimmune disease and/or positive ANA/ENA, yes vs no), active smoking (yes vs no), familiar history of thyroid diseases (yes vs no), age (years) and number of previous pregnancies (N) as covariates.

A regression model including five-knot restricted cubic spline (RCS) was identified as the most informative to describe the relationship between BMI and quantitative TSH by comparing the Akaike information criterion (AIC) and the adjusted R2 of four alternative models (i.e. linear regression, linear regression including three-, four- and five-knot RCS on BMI) with/without the inclusion of additional variables associated with TSH. Variables resulting informative with respect to quantitative TSH distribution from univariate statistical tests (*P* < 0.05) and included in the multivariable models were: familiar history of thyroid diseases (values: no, yes), history of non-thyroidal autoimmune diseases (values: no, yes) and smoking status (values: not active, active). Age (quantitative variable) was not included in the model since it showed no evidence of statistical correlation with TSH (*P* > 0.05). RCS analyses and graphical representations were performed by R version 4.2.2, a free software environment for statistical computing and graphics (www.r-project.org), using functions implemented in the packages called *rms* and *ggplot2*.

A multivariable logistic regression model was designed including a median UtA-PI >95th centile (yes vs no) as dependent variable and TSH ≥4 mU/L (yes vs no), BMI (kg/m^2^), age (years), non-thyroid autoimmunity (i.e. personal history of a non-thyroidal autoimmune disease and/or positive ANA/ENA, yes vs no), familiar history of thyroid diseases (yes vs no), number of previous pregnancies (N) and active smoking (yes vs no) as covariates.

A *P* < 0.05 was considered statistically significant.

## Results

### Clinical characteristics of the study group

The clinical features of the whole study group and of the subgroups are summarized in Table 1.

**Table 1:** Summary of clinical characteristics of the study group and comparison of subjects with a first-trimester TSH value above or below 4 mIU/L. Data are expressed as *n* (%) or as median (25th–75th percentiles) or number (percentage).

	Whole group	TSH		*P*
		<4 mIU/L	≥4 mIU/L	
*n*	2268	2156	112	
Maternal age (years)	32 (29–36)	32 (29–35)	34 (28–37)	0.074
BMI (kg/m^2^)	22.4 (20.6–25.0)	22.3 (20.5–24.9)	23.6 (21.5–29.0)	**<0.001**
Active smokers	207 (9.1%)	205 (9.5%)	2 (1.8%)	**0.006**
Number of previous pregnancies	1 (1–2)	1 (1–2)	2 (1–3)	0.055
Positivity for ANA/ENA screening	100 (4.4%)	79 (3.7%)	21 (18.8%)	**<0.001**
History of NTAID	9 (0.4%)	1 (0.0%)	8 (7.1%)	**<0.001**
Familiar history of thyroid diseases	126 (5.6%)	95 (4.4%)	31 (27.7%)	**<0.001**
Median UtA-PI >95th centile	26 (1.1%)	13 (0.6%)	13 (11.6%)	**<0.001**
Median UtA-PI	1.06 (0.91–1.27)	1.06 (0.90–1.25)	1.42 (1.15–1.70)	**<0.001**

Bold values indicate *P* < 0.05.

NTAID, non-thyroidal autoimmune diseases.

Pregnant women with a TSH above 4 mU/L had a significantly higher BMI, as well as a higher rate of personal history for non-thyroidal autoimmune diseases, of familiar history of thyroid diseases, of positivity for the autoimmunity screening and a lower rate of active smoking. Moreover, women with a TSH above 4 mU/L had higher values of both median UtA-PI and a higher percentage of subjects with a median uterine artery value >95th centile.

Following the WHO definition, 1693 women were classified as normal weight, 435 as overweight and 140 as obese. Table 2 summarizes the main demographic and clinical features of pregnant women stratified according to their BMI. The three groups were similar in terms of age, smoking habit, positivity for ANA and ENA autoantibody screening, familiar history for thyroid diseases and personal history of non-thyroid autoimmune diseases. Median serum TSH was significantly higher in the obese pregnant women when compared to normal weight controls and overweight subjects. Similarly, the percentage of pregnant women having a serum TSH level ≥4 mU/L was significantly higher in the obese subgroup than in the normal weight and overweight one.

**Table 2:** Clinical features of pregnant women according to their BMI. Data are expressed as *n* (%) or as median (25th–75th percentiles) or number (percentage).

	Normal weight	Overweight	Obese	*P*
*n*	1693	435	140	
Maternal age (years)	32 (29–36)	32 (29–36)	32 (28–35.7)	0.937
BMI (kg/m^2^)	21.5 (20.2–22.9)	26.5 (25.7–27.9)*	32.6 (31.2–34.5)*^,^^#^	**<0.0001**
Active smokers	154 (9.1%)	37 (8.5%)	6 (11.4%)	0.577
Number of previous pregnancies	1 (1-2)	2 (1-2)*	2 (1–3)*	**<0.001**
Positivity for ANA/ENA screening	66 (3.9%)	27 (6.2%)	7 (5.0%)	0.105
Personal history of NTAID	7 (0.4%)	1 (0.2%)	1 (0.7%)	0.713
Familiar history of thyroid diseases	88 (5.2%)	24 (5.5%)	14 (10.0%)	0.058
TSH (mIU/L)	1.6 (1.2–2.2)	1.6 (1.2–2.2)	1.8 (1.4–2.4)*^,#^	**0.005**
TSH ≥4 mUI/L	66 (3.9%)	22 (5.1%)	24 (17.1%)*^,#^	**<0.001**
Median UtA-PI >95th centile	16 (0.9%)	5 (1.1%)	5 (3.6%)*^,^#	**0.020**
Median UtA-PI	1.05 (0.90–1.26)	1.09 (0.91–1.30)	1.05 (0.93–1.34)	0.281

Bold values indicate *P* < 0.05.

*P < 0.05 vs normal weight; ^#^ *P* < 0.05 vs overweight.

NTAID, non-thyroidal autoimmune diseases.

### Evaluation of the relationship between BMI values and the risk of having a TSH level above 4 mU/L

A logistic regression model including the main variables correlated with having a TSH level above 4 mU/L was designed, as shown in Table 3. BMI was a significant predictor of having a TSH level above 4 mU/L, independently from non-thyroid autoimmunity, familiar predisposition for thyroid diseases, age, number of pregnancies or smoking.

**Table 3:** Results of the logistic regression model including a TSH ≥4 mU/L (yes vs no) as dependent variable and BMI (kg/m^2^), age (years), number of previous pregnancies (N), non-thyroid autoimmunity (i.e. personal history of a non-thyroidal autoimmune disease and/or positive ANA/ENA, yes vs no), familiar history of thyroid diseases (yes vs no) and active smoking (yes vs no) as covariates.

	OR	95% CI		*P*
		Lower	Upper	
BMI (kg/m^2^)	1.117	1.072	1.165	**<0.001**
Age (years)	1.014	0.972	1.088	0.514
Number of previous pregnancies	1.208	0.986	1.480	0.068
Non-thyroid autoimmunity (yes vs no)	5.896	3.377	10.296	**<0.001**
Familiar history of thyroid diseases (yes vs no)	7.441	4.554	12.159	**<0.001**
Active smoking (yes vs no)	0.191	0.046	0.802	**0.024**

Bold values indicate *P* < 0.05.

### Evaluation of the non-linear relationship between BMI and TSH values considered as continuous variables

**Figure 2:**
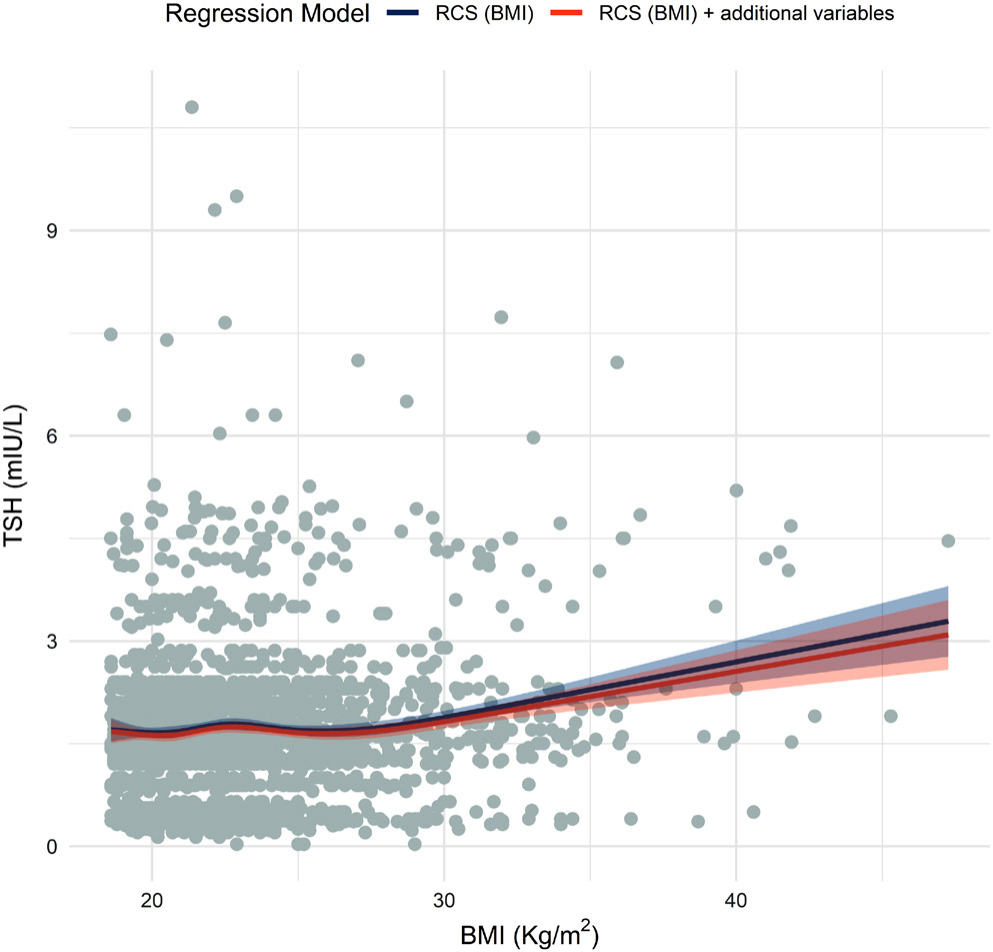
Scatterplot describing the relationship between BMI and TSH as continuous variables. Dots in gray correspond to single observations. The line in blue describes the non-linear relationship between BMI and TSH deriving from the regression model including only BMI, while the line in red describes the non-linear relationship between BMI and TSH deriving from the regression model including additional variables (i.e. holding constant the additional variables values in the model (familiar history of thyroid diseases = no, history of non-thyroidal autoimmune diseases = no, smoking status = not active)). Shaded areas represent 95% confidence intervals. RCS, restricted cubic spline; BMI, body mass index; TSH, thyroid-stimulating hormone.

Regression models including five-knot RCS were fit to investigate the relationship between quantitative BMI and TSH values with and without adjustment for relevant variables. Results shown in Fig. 2 confirm the hypothesis of a non-linear association between BMI and TSH in both unadjusted and adjusted regressions (non-linearity *P* < 0.01). In details, curves show an increasing trend in terms of TSH for BMI values above 31.04 kg/m^2^ (5th knot), with a poorly informative relationship with TSH values below this threshold. In the model including BMI and informative variables, the relationship between TSH and BMI (overall *P* < 0.0001) was independent from familiar history of thyroid diseases and history of non-thyroidal autoimmune diseases, which were associated to significantly higher TSH values (*β* = 0.63, 95% CI = 0.44–0.81, *P* < 0.0001 and *β* = 0.51, 95% CI = 0.30:0.71, *P* < 0.0001 respectively). On the opposite, active smoking status was associated to significantly lower TSH values (*β* = −0.17, 95% CI = −0.31:−0.02, *P* = 0.025).

### Evaluation of the potential impact of a TSH value ≥4 mU/L on UtA-PI according to BMI

To evaluate the impact of increased TSH on early placentation among women with different BMI we compared the values of UtA-PI according to TSH levels above or below 4 mU/L in women with a BMI above or below 30 kg/m^2^.

The results, shown in Table 4, highlight that a TSH value above 4 mU/L at the first trimester was significantly correlated with a higher UtA-PI in women with a BMI both ≥30 kg/m^2^ and below 30 kg/m^2^. The same difference could be seen in terms of increased frequency of a UtA-PI >95th centile. A logistic regression model including the main variables correlated with a higher UtA-PI confirmed that having a TSH level above 4 mU/L was a strong predictor of having a pathologic UtA-PI, independently from BMI (Table 5).

**Table 4:** Comparison of median uterine artery PI (UtA-PI) and percentage of subjects with a median uterine artery values >95th centile between pregnant women with a TSH value above and below 4 mU/L, stratified according to a BMI above or below 30 kg/m^2^. Data are expressed as median (25th–75th percentiles) or as *n* (%).

	TSH		*P*
	<4 mU/L	≥4 mU/L	
BMI <30 kg/m^2^ (*n* = 2128)			
Median UtA-PI >95th centile	11 (0.5%)	10 (11.4%)	**<0.001**
Median UtA-PI	1.06 (0.90–1.25)	1.42 (1.16–1.70)	**<0.001**
BMI ≥30 kg/m^2^ (*n* = 140)			
Median UtA-PI >95th centile	2 (1.7%)	3 (12.5%)	**0.010**
Median UtA-PI	1.03 (0.93–1.24)	1.45 (0.95–1.74)	**0.002**

Bold values indicate *P* < 0.05.

**Table 5:** Results of the logistic regression model including a median UtA-PI >95th centile (yes vs no) as dependent variable and TSH value ≥4 mU/L (yes vs no), BMI (kg/m^2^), age (years), non-thyroid autoimmunity (i.e. personal history of a non-thyroidal autoimmune disease and/or positive ANA/ENA, yes vs no), familiar history of thyroid diseases (yes vs no), number of previous pregnancies (N), active smoking (yes vs no) as covariates.

	OR	95% CI		*P*
		Lower	Upper	
TSH≥4 mU/L	25.174	10.087	62.830	**<0.001**
BMI (kg/m^2^)	1.042	0.962	1.129	0.316
Age (years)	1.051	0.969	1.141	0.228
Non-thyroid autoimmunity	1.662	0.479	5.764	0.423
Familiar history of thyroid diseases	0.509	0.124	2.089	0.349
Number of previous pregnancies	0.171	0.067	0.438	**<0.001**
Active smoking	0.620	0.080	4.829	0.648

Bold values indicate *P* < 0.05.

## Discussion

The results of the present study demonstrate that there is a significant relationship between early pregnancy BMI and TSH serum levels among pregnant women with negative circulating AbTPO. This relationship is independent from other surrogate markers of a higher susceptibility toward autoimmune diseases, such as a familiar history for thyroid diseases, a personal history of non-thyroid autoimmune diseases and a positive screening test for ANA and ENA. The relationship between BMI and TSH levels appears to be non-linear, with a sharp increase in TSH levels among women with a BMI above 30 kg/m^2^. Our results also suggest that having a TSH level ≥4 mU/L may have detrimental effects on pregnancy outcomes both in normal weight and in obese women, since we observed a similar impact of increased serum TSH levels on UtA-PI, an indicator of the quality of early placentation process.

The results of the present study can provide clinically relevant information regarding the management of pregnant women with an increased serum TSH at the beginning of pregnancy. In particular, the notion that BMI in the obese range is significantly related with higher TSH levels is particularly relevant in AbTPO-negative women, since positive AbTPO are one of the most recognized risk factors for increased serum TSH levels during pregnancy. Several previous studies focusing on thyroid function during pregnancy also evaluated the possible relationship between TSH levels and maternal BMI at the beginning of pregnancy, with contrasting results. While some authors highlighted a positive correlation between TSH and BMI during the first trimester of pregnancy (16, 17, 18, 19, 20), this phenomenon was not observed in other studies (23, 24, 25). These discrepant results could be due to the fact that some studies included both autoantibody positive and negative women (16, 19, 23, 24, 25), while other focused on autoantibody negative ones (20), or did not evaluate thyroid autoantibodies (18).

The pathophysiology of the increase in TSH values experienced by women living with obesity is still uncertain. While autoimmunity is the most widely recognized cause of subclinical hypothyroidism, both in pregnant and non-pregnant subjects, several other causes have been described, including the presence of laboratory interferences, the presence of TSH genetic variants, iron and iodine deficiency and, also, obesity (14). Indeed, a hyperthyrotropinemia in absence of serological and ultrasonographic evidence of thyroid autoimmunity has been extensively described in non-pregnant subjects living with obesity (14, 15). It can be hypothesized that a similar mechanism could lead to an increase in TSH levels also in pregnant women living with obesity. To confirm this hypothesis, we designed a logistic regression model, including presence of non-thyroid autoimmunity (i.e. anamnestic record of non-thyroidal autoimmune diseases and/or positivity for ANA and ENA screening) familiar history of thyroid diseases, age, number of pregnancies and active smoking as covariates. The rationale of including ANA/ENA positivity as a covariate in our analysis stems from the well known clustering of thyroid autoimmunity with other non-thyroidal autoimmune diseases. Indeed, rheumatic and thyroid autoantibodies during pregnancy are closely associated (33). In a previous study by our group, AbTPO negative women with a TSH >4 mIU/L had a higher prevalence of ANA/ENA positivity and of family history of thyroid diseases when compared to healthy controls (5). In this context, ANA/ENA positivity, the presence of other autoimmune diseases and the family history of thyroid diseases can be all considered as surrogate markers of an autoimmune pathogenesis of TSH increases in patients without the most used marker of thyroid autoimmunity, i.e. TPOAb. The finding that TSH increases in pregnant women with a higher BMI is independent from these confounding factors strengthens the hypothesis that this phenomenon is not due to a higher rate of serum-negative thyroid autoimmunity in obese pregnant women but rather to a direct effect of increased adiposity on TSH levels.

Some authors suggest that one of the most intriguing mechanisms for explaining obesity-related hyperthyrotropinemia is related to leptin, an adipocyte-derived hormone, and a key regulator of energy expenditure and food intake (34). Leptin and the hypothalamus–pituitary–thyroid (HPT) axis have a bidirectional interaction, since leptin receptors are expressed on TRH-secreting neurons in the paraventricular nucleus of the hypothalamus (35) and TSH receptors are expressed on adipocytes (36). The downregulation of the HPT axis during food deprivation is probably mediated by the concomitant reduction in circulating leptin. Moreover, *in vivo* administration of recombinant human TSH at supra-physiological doses can induce the release of small amounts of leptin (15). Notwithstanding the large literature on this topic, the role of leptin signaling in thyroid dysfunction, and particularly in obesity-related hyperthyrotropinemia, is still highly controversial (37). This mechanism could be particularly relevant during pregnancy, since leptin concentration are strongly correlated with pre-pregnancy BMI (38). Moreover, leptin plays a key role in the immune mechanisms regulating implantation, trophoblastic invasion and placental angiogenesis (39), and persistently high levels of leptin in obese women are predictive of preeclampsia (40). Nevertheless, no study has ever specifically evaluated the role of leptin in determining an increase in TSH levels during pregnancy.

Our results also suggest that the relationship between BMI and thyroid function could be not linear, with a more sharp increase with BMI values above 31 kg/m^2^.

The results of the present study do not allow to draw conclusions on the impact of the increased serum TSH observed in pregnant women living with obesity on obstetric and perinatal complications since the observation was limited to the first trimester. However, when considering the first-trimester UtA-PI as a surrogate early indicator of abnormal placentation and thus as a predictor of subsequent risk of developing pregnancy complications, our findings indicate that having a TSH level ≥4 mU/L, even in absence of TPOAb positivity, is a significant risk factor for an increased UtA-PI, confirming our previous data (5). The results of the present study show that having a TSH level ≥4 mU/L was significantly correlated with having pathological UtA-PI both in pregnant women with a BMI in the normal/overweight range and in the frankly obese ones. These data could indicate that the raised TSH experienced by women with a BMI above 30 kg/m^2^ would not be only a non-significant biochemical alteration secondary to the high BMI, but it could have a clinical impact on the placentation process. This information is particularly relevant since obesity *per se* has a well recognized impact on several obstetrical outcomes that have an abnormal placentation as a pathogenetic mechanism, including pre-eclampsia (41) and preterm birth (42).

As a further relevant finding, our findings confirm previous data indicating that active smoking during pregnancy was more frequent among women with lower TSH values. This finding is in line with previous reports both in non-pregnant subjects (43) and during pregnancy (44, 45). A higher T3/T4 ratio was also observed in the latter case (22). One possible explanation of this finding is smoking-mediated increase in deiodinase activity, paraleled with an increased activation of the sympathetic nervous system, although the exact implications of this phenomenon are still unknown.

Our study has some limitations. Systematic measurement of FT4 and FT3 was not available for the majority of included subjects, so we could not evaluate the relationship between maternal obesity and circulating free thyroid hormones or FT3/FT4 ratio. Indeed, a finding commonly reported during pregnancy is a reduction in FT4 levels and a higher FT3/FT4 ratio (46, 47). Although the mechanism is not completely understood, obesity could increase deiodinase activity, possibly through the action of leptin, resulting in lower FT4 and higher FT3 (47), especially in areas characterized by iodine deficiency (48). This phenomenon would not be free of clinical repercussions, since recent studies suggest a U-shaped relationship between gestational free thyroid hormone levels and risk of preeclampsia (49).

The fact that TgAb were not measured in this cohort could have led to the missed diagnosis of several pregnant subjects with thyroid autoimmunity. Indeed, a recent study suggested that measuring TgAb could be particularly helpful in the differential diagnosis of hyperthyrotropinemia among subjects living with obesity (50). Moreover, evaluation of body composition and adiposity would add some relevant information on the impact of BMI on thyroid function. Lastly, the cross-sectional design of the study does not allow to elucidate if there is a cause–effect relationship between thyroid function and BMI. Nevertheless, the enrollment of high number of subjects with negative AbTPO allows us to draw some firm conclusions on the relationship between BMI and thyroid function during pregnancy.

In conclusion, the results of this study show a significant correlation between obesity and TSH serum levels above the cut off of 4 mIU/L in AbTPO-negative pregnant women, independently from the other risk factors usually associated to subclinical hypothyroidism during pregnancy. According to our analysis, the risk of higher TSH levels became evident with BMI indicative of class I obesity (BMI >30 kg/m^2^). The increase of TSH levels could be clinically relevant as suggested by its association with the rate of abnormal UtA-PI detected during the first trimester.

Prospective studies are needed to better elucidate the role of hyperthyrotropinemia related to obesity on obstetrical and neonatal outcomes.

## Declaration of interest

The authors declare that there is no conflict of interest that could be perceived as prejudicing the impartiality of the study reported.

## Funding

This work was partially supported through the Ricerca Corrente funding of the Italian Ministry of Healthhttp://dx.doi.org/10.13039/100009647. FC and FM were partially supported by the National Recovery and Resilience Plan (NRRP), Mission 4 Component 2 Investment 1.3 – Call for proposals No. 341 of March 15, 2022, of Italian Ministry of University and Research funded by the European Union – NextGenerationEU; Award Number:Project code PE00000003, Concession Decree No. 1550 of October 11, 2022, adopted by the Italian Ministry of University and Research, CUP D93C22000890001, Project title ‘ON Foods – Research and innovation network on food and nutrition Sustainability, Safety and Security – Working ON Foods’.

## Author contribution statement

Conceptualization: LC, FB, FM. Methodology: LC, FB, MR, FM. Data curation: LC, IDM, CB, FC, MT. Writing: LC, FR, MT. Original draft preparation: LC, FR, MT. Visualization, Investigation: LC, FB, IDM, CB, FM. Supervision, Reviewing and Editing: FB, MR, AS, FM.
